# Importance of software version for measurement of arterial stiffness: Arteriograph as an example

**DOI:** 10.1371/journal.pone.0197019

**Published:** 2018-05-21

**Authors:** Margareta Ring, Maria J. Eriksson, Gunnar Nyberg, Kenneth Caidahl

**Affiliations:** 1 Department of Molecular Medicine and Surgery, Karolinska Institutet, Stockholm, Sweden; 2 Department of Clinical Physiology, Karolinska University Hospital, Solna, Stockholm, Sweden; 3 Department of Molecular and Clinical Medicine, Institute of Medicine, Sahlgrenska Academy, University of Gothenburg, Gothenburg, Sweden; University of Colorado Denver School of Medicine, UNITED STATES

## Abstract

**Background:**

Current guidelines recommend the measurement of arterial stiffness in terms of aortic pulse wave velocity (PWV) as an important cardio-vascular risk marker. Both aortic PWV and the aortic augmentation index (AIxao) can be measured using different techniques, e.g., the Arteriograph and SphygmoCor. A new version of the software for the Arteriograph (v. 3.0.0.1, TensioMed, Budapest, Hungary; Arteriograph II) is now available. We wanted to determine whether this improved software differs from the previous version (Arteriograph v. 1.9.9.12; Arteriograph I). We compared the estimated aortic PWV (ePWVao) and AIxao measured with both versions of Arteriograph software and analysed the agreement of these values with those measured by SphygmoCor (v. 7.01, AtCor Medical, Sydney, Australia).

**Methods:**

Eighty-seven subjects without known cardio-vascular disease (23 men and 64 women) aged 54.2 ± 8.7 years (mean ± standard deviation; range 33–68 years) were included in the study. Estimated PWVao and AIxao were measured by both Arteriograph and SphygmoCor. We compared Arteriograph I and Arteriograph II with each other and with SphygmoCor.

**Results:**

Estimated PWVao measured by Arteriograph II was lower than that measured by Arteriograph I, while the AIxao was higher. Divergence in ePWVao values was especially noted above 9 m/s. Estimated PWVao measured by Arteriograph II (7.2 m/s, 6.6–8.0 [median, 25th–75th percentile]) did not differ from that measured by SphygmoCor (7.1 m/s, 6.7–7.9 [median, 25th–75th percentile]). However, the AIao measured by Arteriograph II was significantly higher (*P* < 0.001).

**Conclusion:**

Regularly upgraded software versions resulting from continuous technical development are needed for quality improvement of methods. However, the changes in software, even if the basic patented operational algorithm has not changed, may influence the measured values as shown in the present study. Therefore, attention should be paid to the software version of the method used when comparing arterial stiffness results in clinical settings or when performing scientific studies.

## Introduction

Arterial stiffness, measured as aortic pulse wave velocity (PWVao) is an important marker of cardio-vascular risk and has been reported to be an independent predictor of cardio-vascular morbidity and mortality [1]. Carotid-femoral PWV is considered as a clinically accessible surrogate measure of arterial stiffness [2]. Various types of equipment have been introduced for pulse wave in clinical research. An early such was Complior, with automatic PWV measurement for assessment of arterial distensibility [3]. Subsequently, SphygmoCor (SC) was developed, first for the purpose of measuring aortic augmentation index, later modified to measure central pulse wave velocity [4, 5]. The Arteriograph (AG) technique was introduced to be an easy to use method, possible to be applied in general clinical practice. Thus, different techniques measuring arterial stiffness have been available recent years, and reference values may differ between techniques. AG relies on an oscillometric method, while SC is based on a tonometric technique, and both have been validated and compared with invasive techniques [6, 7].

We have previously demonstrated a good reproducibility for the AG and SC techniques, but some differences between techniques such as higher PWV values for women, highlighting the necessity to use the same type of equipment throughout scientific studies [8].

Although measuring arterial stiffness variables, previous methodological studies have shown some divergence between the values obtained by the AG and SC techniques [8–13]. This knowledge has implications when comparing results and designing new research projects.

Continuous software development and improvement within the same measurement technique usually lead to updates to new versions for the same technique. These updates constitute another possible source of variation in results. Whether changes between different software versions influence the obtained values is, however, unknown since such studies are lacking.

Therefore, the aim of this study was to investigate whether values of estimated PWVao (ePWVao) and the aortic augmentation index (AIxao) recalculated by a newer software version from original raw data differed from those obtained using an earlier version of the software. For this purpose, we compared results from same raw data applying two versions of AG software introduced between 2006 and 2014.

We also wanted to study the agreement of ePWVao and AIxao values obtained by the new version of AG software with those obtained using SC.

## Methods

Eighty-seven non-smoking subjects (23 men and 64 women) without known cardio-vascular disease, age range 33–68 years (mean ± standard deviation, 54.2 ± 8.7 years), were included in the study. No subject had diabetes mellitus (P-Glucose, 5.0 (4.7–5.2) mmol/L, median (25^th^-75^th^ percentile)), renal disease, known hypertension or was taking any medication affecting the cardio-vascular system. The study group was recruited from a population of 96 participants (48 healthy subjects and 48 patients with mild primary hyperparathyroidism) in a study previously described in detail [14, 15]. Only subjects with acceptable pulse wave recordings from both SC and AG (N = 87) were included in the present study.

All subjects were studied after an overnight fast and after no caffeine intake from midnight the night before examination. Pulse wave recordings were acquired by SC followed by AG after approximately 60 min rest in the supine position in a quiet room. Because the SC method involves minimal influence on the arm arteries, while the AG investigation involves periods of supra systolic pressure, the same sequence of investigations was followed for all subjects.

All measurements were performed by the same experienced investigator. Each participant provided written informed consent to participate in the study, which was approved by the Regional Ethics Review Board in Stockholm, Sweden.

### Arteriograph

With the AG technique (Arteriograph; TensioMed, Budapest, Hungary), a cuff is placed on the patient’s upper arm; pressure variations in the arm influence pressure receptors in the cuff and are transferred via an infra-red port to a computer. The systolic blood pressure (SBP) and diastolic blood pressure (DBP) in the upper arm were measured at the first cuff inflation, Table 1. Then, during a second inflation at 35 mmHg above the SBP, the pressure pulse configuration was recorded. The basis of this occlusion technique generates two systolic peaks. The first peak (P1) is created by the ejection of the blood volume from the left ventricle (LV) into the aorta. The second peak (P2) is created by the reflected wave from the periphery (average assumed to be around the aortic bifurcation). An early reflected pulse wave in the central aorta is caused by increased arterial stiffness [16]. The AIxbr (augmentation index brachialis) was calculated as 100*(P2–P1)/pulse pressure (PP). The AIxao was calculated from its relationship with AIxbr from empiric data, using their close relationship [6]. The return time (RT) is the difference (ms) between the first (P1) and the reflected (P2) systolic waves and is related to the stiffness of the aorta. The aortic distance was measured as the distance from the jugular fossa to the symphysis (Jug–Sy). Estimated PWVao was then calculated as the Jug–Sy distance (m) divided by RT/2 (s). Estimated PWVao and AIxao were measured from the right upper arm and are presented as the mean value of two recordings, chosen from recordings with the lowest standard deviation (SD). The mean and SD values were based on every heart beat during a period of 8 s. At initial recordings, the software Arteriograph version v. 1.9.9.12 (AG I) was used for calculations. The same variables were now recalculated from the same raw data using the more recent software version v. 3.0.0.1 (AG II).

**Table 1:** Vascular data measured by SphygmoCor and Arteriograph.

N = 87	SC	AGI	AGII	P	P	P
	v.7.01	v.1.9.9.12	v.3.0.0.1	SC-AGI	SC-AGII	AGI-AGII
**ePWVao, m/s**	7.1 (6.7–7.9)	7.5 (6.9–8.8)	7.2 (6.6–8.0)	<0.01	NS	<0.001
**AIxao, %**	29.0 (18.0–36.5)	32.3 (22.0–40.2)	36.2 (25.4–46.3)	<0.001	<0.001	<0.001
**TT, RT/2, ms**	65.5 (57.3–71.2)	68.5 (57.0–76.0)	71.3 (63.8–80.0)	NS	<0.001	<0.001
**Distance, cm**	46.5 (43.0–49.0)	52 (49.0–54.0)	-	<0.001	-	-
**SBP, mmHg**	117.0 (109–129)	116.0 (110–124.5)	-	NS		
**DBP, mmHg**	76.0 (69.0–81.0)	75.0 (69.0–81.0)	-	NS		
**HR, bpm**	56.0 (52.0–61.0)	55.5 (51.0–61.0)	-	NS		

Data are presented as median (25^th^-75^th^ percentile), SC = SphygmoCor, AG = Arteriograph, ePWVao = estimated aortic pulse wave velocity, AIxao = Aortic augmentation index, TT = Transit time by SphygmoCor, RT = Return time by Arteriograph, SBP = Systolic blood pressure, DBP = Diastolic blood pressure, HR = Heart rate.

### SphygmoCor

Arterial pulse waves measured by SC (v. 7.01; AtCor Medical, Sydney, Australia) were registered with a single high-fidelity tonometer (SPT-301B; Millar Instruments, Houston, TX, USA). The tonometer connected to the SC equipment was gently pressed onto the artery of interest. The arterial pulse waves were processed by the system software, and the corresponding aortic pressure waveform was generated from the radial artery waveform using a validated transfer function [4, 5]. The right radial pulse wave was calibrated against the brachial blood pressure, which was determined as the mean of two measurements on the right arm, obtained just before start of the examination, Table 1. The AIxao was defined as the difference between the first (P1) and the second (P2) peaks of the central aortic waveform, expressed as a percentage of PP. For ePWVao an electrocardiogram (ECG) was connected and the transit time (TT) between pulse arrival at the left common carotid artery and the right femoral artery was calculated using the R-wave of the ECG as a reference. A measuring tape was used to estimate the distance that the pulse travelled. The jugulum–femoral length was obtained as the jugulum-to-umbilicus plus umbilicus-to-right femoral artery distances. The carotid–jugulum distance was subtracted from the jugulum–femoral distance; L = (jugulum–femoral)–(carotid–jugulum). PWVao = L/TT m/s. The PWVao recordings were chosen from the waveforms with the lowest SD, and AIxao from the waveforms within the limits of the current quality control settings and a quality index above 0.85. Estimated PWVao and AIxao are presented as the mean values of two recordings.

### Blood pressure

The arterial blood pressure was measured in both arms, after 30 min of supine rest, using a digital automatic blood pressure monitor (Omron M7; Omron Healthcare Co., Ltd., Kyoto, Japan). The mean values of the SBP and the DBP in both arms were calculated.

### Statistics

Statistical analyses were performed using Statistica (v. 9.0; Statsoft, Inc., Tulsa, OK, USA). Data are expressed as mean ± SD or median and 25^th^–75^th^ percentile. The tests were two-tailed and a *P*-value of < 0.05 was considered significant. The Wilcoxon signed-rank sum test was used to test differences between the methods and software versions. The Bland–Altman test was used to evaluate the variability between the software versions and methods [17]. In our previous study with a similar design, comparing AG with SG a number of 63 patients was adequate to guarantee a power level of 80% at a confidence level of 95% [8]. In this study we included 87 individuals, in whom AG I and SC were originally used, and data by AG II were obtained by recalculation using raw data from AG I. Data were analysed under supervision of a professional statistician.

## Results

The mean body mass index (mean ± SD) of the participants was 23.6 ± 3.16 kg/m^2^, SBP 122 ± 14 mmHg and DBP 77 ± 8 mmHg. Vascular data obtained using SC, AG I and AG II are presented in Table 1.

We found significant differences between AG I and AG II for PWVao and AIxao values (all *P* < 0.001). No significant differences were seen for ePWVao measured by SC (7.1, 6.7–7.9 m/s) and AG II (7.2, 6.6–8.0 m/s). However, AIxao measured by SC (29.0, 18.0–36.5%) was lower compared with that measured by AG II (36.2, 25.4–46.3%) (*P* < 0.001).

**Fig 1:**
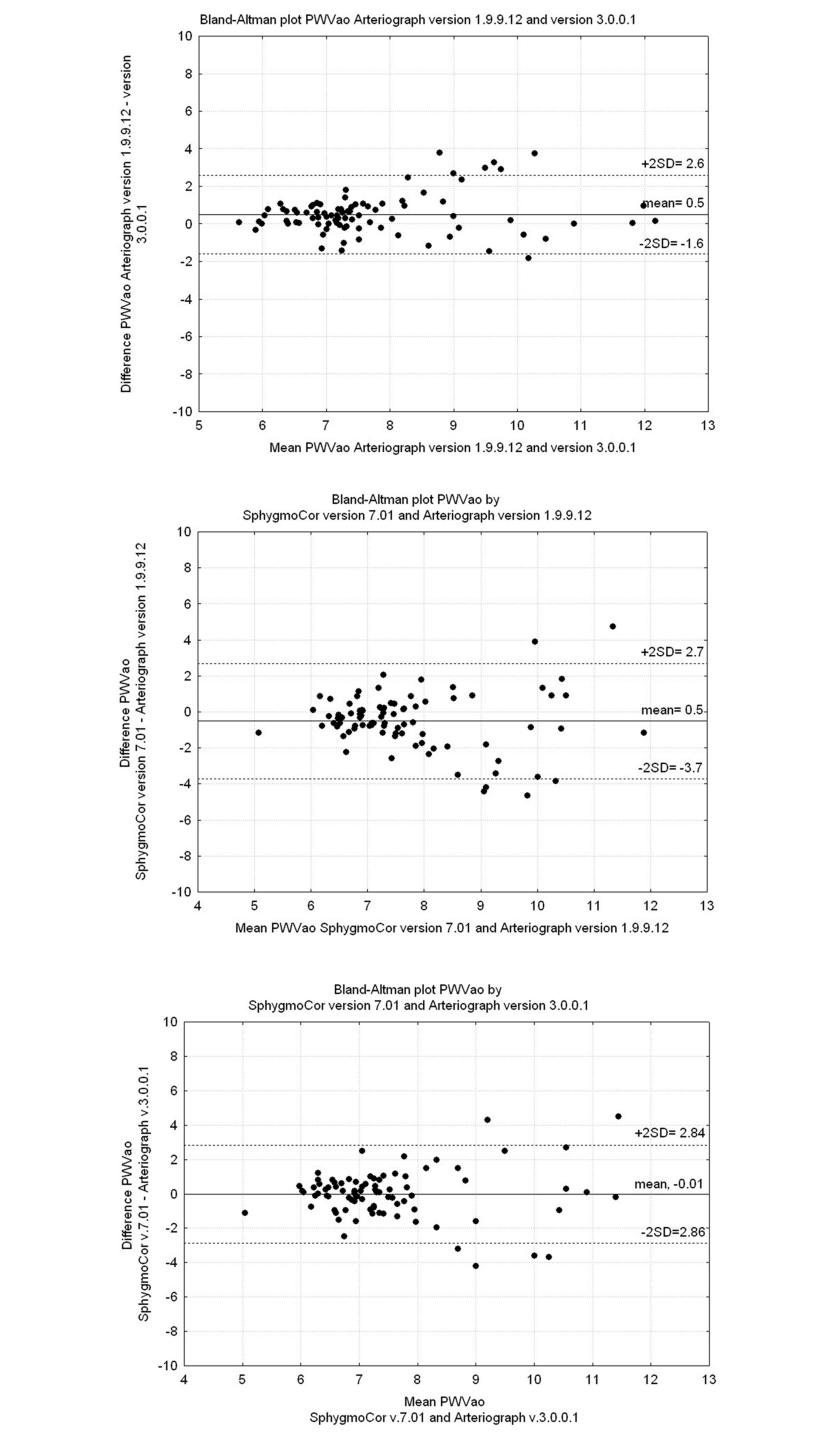
Bland-Altman plots showing PWVao. (Upper panel) Arteriograph v. 1.9.9.12 and v. 3.0.0.1. (Mid panel) SphygmoCor v. 7.01 and Arteriograph v. 1.9.9.12. (Lower panel) SphygmoCor v. 7.01 and Arteriograph v. 3.0.0.1.

**Fig 2:**
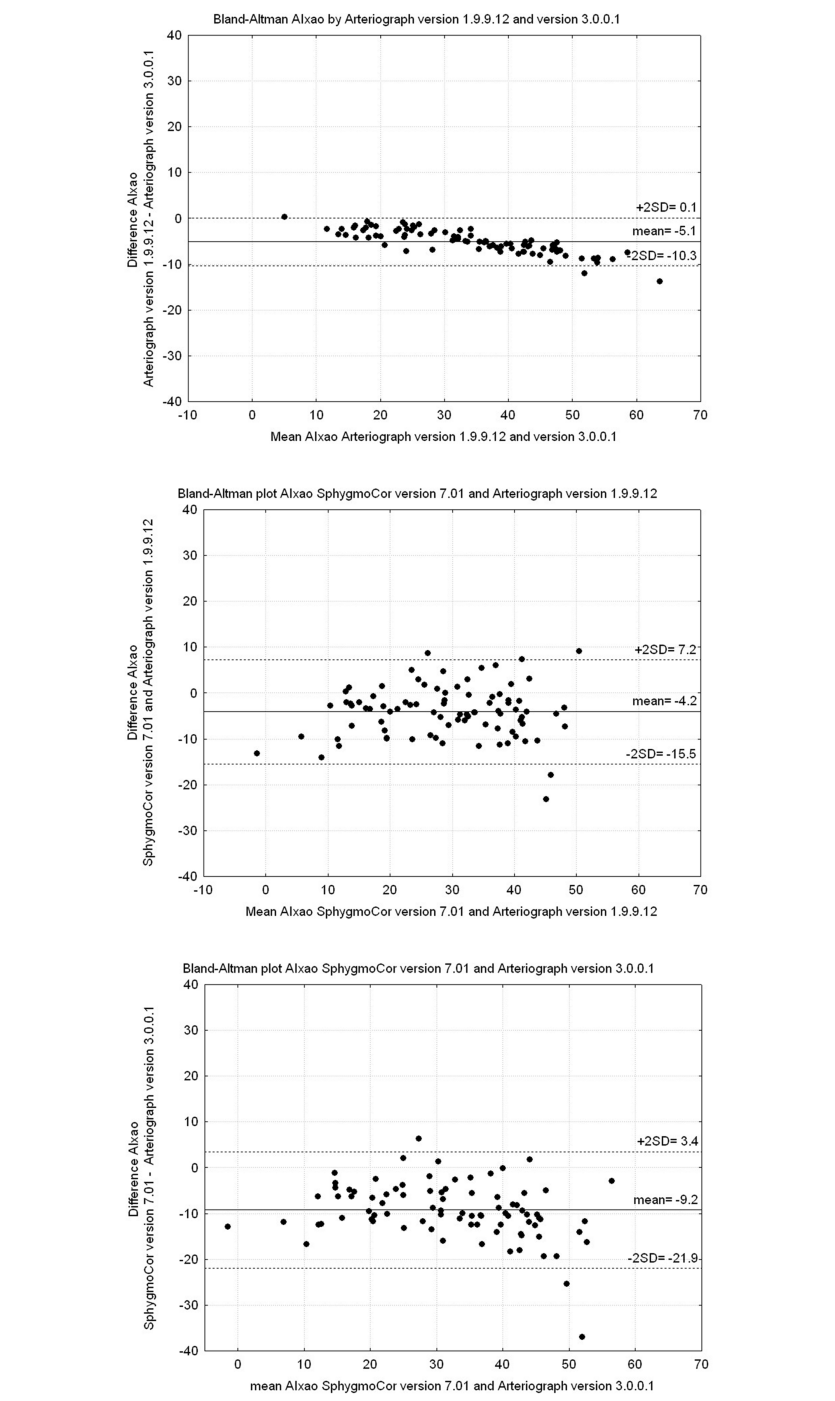
Bland-Altman plots showing AIxao. (Upper panel) Arteriograph v. 1.9.9.12 and v. 3.0.0.1. (Mid panel) SphygmoCor v. 7.01 and Arteriograph v. 1.9.9.12. (Lower panel) SphygmoCor v. 7.01 and Arteriograph v. 3.0.0.1.

The Bland–Altman plots shown in Figs 1 and 2 illustrate the agreement between SC, AG I and AG II for ePWVao and AIxao. Fig 1A illustrates the difference between ePWVao values measured by AG I and AG II. We found that the values were more divergent above 9 m/s. This was also seen in the Bland–Altman plots shown in Fig 1B (SC vs. AG I) and 1C (SC vs. AG II). As illustrated in Fig 1C, the mean difference in ePWVao between SC and AG II showed even better agreement, but the scatter is wide. AIxao measured by AG II showed significantly higher values compared with those measured by AG I (Fig 2A). The agreement of AG with SC was not improved by using AG II (Fig 2B and 2C).

## Discussion

To our knowledge, software versions within methods for measuring arterial stiffness have not been studied and compared. In this study we compared ePWVao and AIxao calculated by AG I software v. 1.9.9.12 with the recalculated values obtained using AG II, an updated version (v. 3.0.0.1) of the software. We also compared these values with those obtained using SC. Our main findings were that on average, ePWVao values obtained using the updated software version AG II were closer to the values obtained using SC than those obtained using AG I, but the scatter was not improved. Furthermore, AIxao values obtained using AG II were significantly higher than those obtained using AG I, and differed even more from the values obtained by SC. This difference might be caused by the new algorithms used in the updated AG II. The return time, RT/2, calculated with AG II was higher than that calculated by AG I; this leads to a decreased ePWVao and better agreement with values obtained using SC. Increased PWVao has been reported to be associated with a higher incidence of cardio-vascular mortality, stroke and coronary heart disease events in older adults [18], and prevention of age-associated arterial stiffening might improve the health of this age group. Logistically, the evaluation of arterial stiffness is important to allow for adequate distribution of preventive measures [19, 20], and we need reliable methods for its measurement. Earlier studies showed that PWVao and AIxao values differ depending on which technique is used [8–13, 21]. Reference values for pulse wave velocity have been published [22]. When using different techniques for measures of arterial stiffness it may be important to identify and use separate reference values for each method. Our results underline that detailed information about equipment used in research, especially when reference values are reported, should be described. Here, we identify a new problem, showing differences in the arterial stiffness values obtained using two software versions for a single method, Arteriograph. We also compared the values obtained using these two software versions with those obtained using SphygmoCor.

Besides software related factors that can cause errors or methodological discrepancies, there are known limitations when collecting pulse waves from carotid and femoral arteries. Obesity may cause a difficulty obtaining an adequate signal in some individuals and may also cause errors in distance measurement and estimation of transit length. The distance should be measured precisely, because small inaccuracies in centimeter may influence the absolute value of PWV. For SphygmoCor, the shorter the distance is between two recordings sites, the greater the absolute error in determining the transit time [2]. The same risk applies for the distance estimation used with the Arteriograph.

The primary determinant of augmentation index is central pulse pressure, which was not measured in this study, and is not directly reflected in peripherally measured blood pressure. Estimation of central BP through transformation algorithms therefore constitutes a potential source of error. Peripherally measured SBP is higher than aortic (central) SBP in young healthy individuals, while the DBP and mean blood pressure remain almost steady throughout the arterial tree [23]. The difference in SBP can reach up to 20 mmHg or more and is known as PP amplification, which is considered to be a result of the pressure wave reflection along the vascular bed and the arterial stiffness gradient [24]. Wilkinson et.al. reported that there is less amplification of the pressure waveform as it travels from the aorta to the brachial artery in older subjects, because of increased early wave reflection and augmentation of central systolic pressure and PP [25]. Besides PP, AIx is dependent on reflection site, influenced by aortic length, varying with height and age.

In this study we compared the two most frequently used pulse wave techniques for estimation of arterial stiffness. Recently imaging techniques, such as magnetic resonance imaging have emerged that allow evaluation of arterial stiffness and may become a future reference method [26]. Reproducibility of PWV measurements with phased contrast magnetic resonance (PCMR) and applanation tonometry (SphygmoCor) has been reported [27]. These authors found no significant differences in PWV values between the two techniques. Further, they stated that “all techniques measure surrogates of pulse wave velocity. Even intravascular pressure measurements are not standardized due to the variety of methods used to determine the transit time of the pulse wave”.

The results from our investigation should encourage further studies on variability of PWVao and AIxao measurements between different software versions of the other methods available. This would apply even for “reference” methods. From clinical and scientific communities there are also expectations on the manufacturers to provide detailed information on the characteristics of new software upgrades and possible consequences for systematic changes in measured values.

## Limitations

An advantage of the present study was the possiblity to use the same raw data for calculations by two software versions for the Arteriograph. We did not have the possibility to do similar recalculations and compare software versions for SphygmoCor.

## Conclusion

Regularly upgraded software versions resulting from continuous technical development are needed for quality improvement of methods. However, the changes in software, even if the basic patented operational algorithm has not changed, may influence the measured values as shown in the present study. Therefore, attention should be paid to the software version of the method used when comparing arterial stiffness results in clinical settings or when performing scientific studies.

## Supporting information

S1 File Data supporting the underlying findings described within the manuscript. (XLSX)
